# Agar-based optical sensors for electric current measurements

**DOI:** 10.1038/s41598-023-40749-7

**Published:** 2023-08-19

**Authors:** Eric Fujiwara, Lidia O. Rosa, Hiromasa Oku, Cristiano M. B. Cordeiro

**Affiliations:** 1 School of Mechanical Engineering, University of Campinas, Campinas, 13083-860 Brazil; 2 Faculty of Informatics, Gunma University, Kiryu, 376-8518 Japan; 3 Institute of Physics, University of Campinas, Campinas, 13083-859 Brazil

**Keywords:** Applied optics, Other photonics

## Abstract

Biodegradable optical waveguides are breakthrough technologies to light delivery and sensing in biomedical and environmental applications. Agar emerges as an edible, soft, low-cost, and renewable alternative to traditional biopolymers, presenting remarkable optical and mechanical characteristics. Previous works introduced agar-made optical fibers for chemical measurements based on their inherent response to humidity and surrounding concentration. Therefore, we propose, for the first time, an all-optical, biodegradable electric current sensor. As flowing charges heat the agar matrix and modulate its refractive index, we connect the optical device to a DC voltage source using pin headers and excite the agar sample with coherent light to project spatiotemporally deviating speckle fields. Experiments proceeded with spheres and no-core fibers comprising 2 wt% agar/water. Once the increasing current stimulates the speckles’ motion, we acquire such images with a camera and evaluate their correlation coefficients, yielding exponential decay-like functions whose time constants provide the input amperage. Furthermore, the light granules follow the polarization of the applied voltage drop, providing visual information about the current direction. The results indicate a maximum resolution of $\sim$0.4 $\upmu$A for electrical stimuli $\le$ 100 $\upmu$A, which fulfills the requirements for bioelectrical signal assessment.

## Introduction

Optical devices manufactured with degradable materials emerge as promising candidates to fulfill the growing demand for biocompatible and green technologies. Implantable waveguides enable light-driven assessment and actuation in healthcare, imaging, drug delivery, and optogenetics^1–5^, ensuring gradual absorption by the organism after its use. Nowadays, waveguides made of silk fibroin^6^ and biopolymers like cellulose^7^, alginate^8^, citrate^9^, polycaprolactone^10^, and poly(d,l-lactic acid)^11^ are available to replace typical glass and plastic optical fibers by attaining tolerable transmission losses. However, most of these approaches depend on relatively expensive precursors and elaborated fabrication routes.

In this context, agar obtained from red algae arises as an edible and renewable alternative for conceiving optical components and waveguides. Agar contains the polysaccharides agarose and agropectin in its composition, the former instituting its gelling ability. This material exhibits singular features like moldability, flexibility, chemical stability, gelation at low temperatures, and thermoreversibility^12–14^. Besides, the mechanical and optical properties of bulk samples (rigidity, refractive index, transparency, etc.) are tailorable by choosing the chemical composition of the agar solution. Emblematic applications of this gel-like material encompass artificial tissues, bioplastics, and growth media for microorganisms^13,14^.

Despite its traditional uses in the food industry and biochemical analysis, scarce literature covers optical devices made of agar. For example, Oku *et al.* developed edible lenses and retroreflectors to create virtual reality setups in aliments^15,16^. Manocchi *et al.* created a planar waveguide comprising agar and gelatin layers, anticipating further use as an implantable biochemical monitor^17^. Jain *et al.* introduced a rectangular waveguide integrated into a microfluidic system for cell immobilization and imaging purposes, achieving optical losses of $\le 13$ dB/cm^18^. Lastly, our group demonstrated a structured optical fiber made of agar comprising a solid core surrounded by air holes. The experiments evaluated optical losses (3.3 dB/cm) and investigated possible applications in chemical sensing^19^.

Agar is intrinsically responsive to the surrounding medium’s concentration, temperature, humidity, and pH, making it eligible for detecting diverse physical and biochemical parameters. Apart from the usual setups comprising glass optical fibers coated with hydrogel films^20–22^, an agar waveguide may absorb or expel water droplets due to the swelling and syneresis mechanisms^23^, respectively, producing geometrical changes that disturb the transmitted light^19^. Furthermore, one may adjust the refractive index of the bulk sample by adding sugar or glycerol to the agar solution, enhancing its sensitivity to liquids flowing outside the fiber or internally through the holey structure^19^.

Even though most of the available optical sensors focus on chemical measurements, agar also exhibits remarkable electrical properties, as evidenced by electromembranes for phase extraction^14^, brain tissue phantoms^24^, impedance-based imaging^25^ and spectroscopy^26^. The electrical conductivity typically arises from the mineral contents in the agar powder and water, increasing with the temperature and the quantity of dissolved salts^27^. Indeed, while the aqueous phase dictates the agar bulk conductivity, adding ionic compounds (like NaCl, NaN$_{3}$, and KCl dissolved in water) to the mixture may conveniently increase its response to electrical stimuli^28–30^. Nevertheless, despite the several uses for membranes and phantoms, no studies exploiting the agar conductivity for current sensing appeared in the literature until a recent work in which we demonstrated the optical modulation of an agar waveguide via electrical input^31^.

Therefore, this paper explores such a phenomenon and proposes agar-based optical sensors for assessing electric currents wherein charges flowing across the material produce temperature deviations that modify its refractive index distribution. Thus, we excite the optical device with coherent light to analyze the dynamic response of the output speckle field, then retrieve the magnitude and direction of the applied stimuli. Experiments validate this sensing principle for bulk agar spheres and no-core optical fibers, yielding reliable results for currents $\le 100$ $\upmu$A.

Apart from the typical optical fiber sensors designed to operate in the A to kA range^32–36^, the ability to detect subtle electric signals inspires possible applications in biomedical setups, like the assessment of neural or muscular responses for predicting dysfunctions and establish assistive human-computer interfaces^37,38^. The soft, bioresorbable optical sensor may replace widespread electronic transducers and take advantage of the fiber sensors’ characteristics, such as flexibility and multiplexing capability. For instance, a launching dielectric fiber may insulate the agar probe from the interrogation system and ensure passive, robust analyses. To the best of our knowledge, this is the first demonstration of an all-optical current sensor based on the electric stimulation of a biodegradable waveguide.

## Results

**Figure 1:**
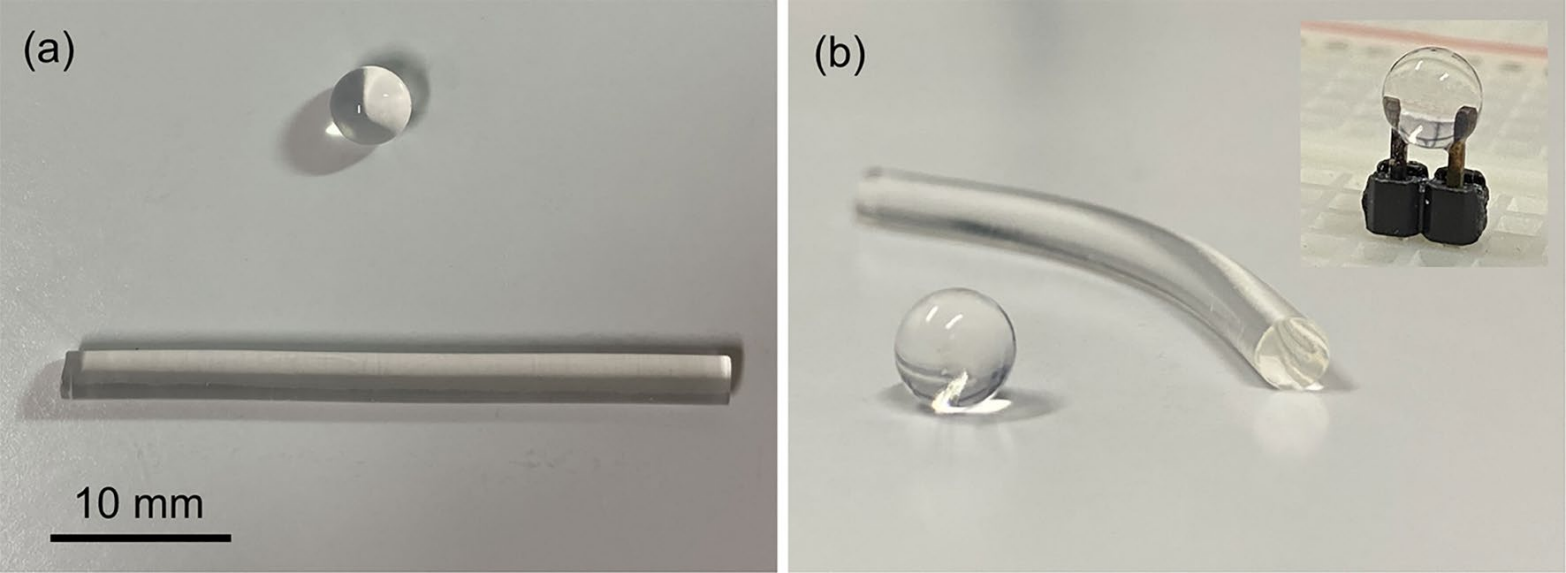
Sphere and no-core optical fiber made of 2 wt% agar: (**a**) top and (**b**) side views (inset: agar sphere coupled with the pin header connectors). The diameters of sphere and fiber are $\sim$4 mm and $\sim$2.5 mm, respectively.

Initial experiments evaluated the optical response of transparent spheres comprising 2 wt% agar concentration and an average diameter of 4 mm, as shown in Fig. 1, subjected to direct currents. Pin headers connect the samples to a breadboard for applying electrical stimulation without disturbing the optical device. Although the transmission in the visible/near-infrared range improves by reducing the agar content, concentrations of 2 wt% are preferable to ensure mechanical strength and chemical stability at room temperature^19^.

A launching multimode fiber (MMF) illuminates the spheres with coherent light to generate output speckle patterns, as the agar works as a random scattering medium^39^. Meanwhile, the voltage source regulates the direct current *i* flowing through the agar samples and promotes dynamic changes in the speckle field distribution. The tests proceeded for $0 \le i \le 104$ $\upmu$A with the CCD camera recording 500 sequential specklegram frames ($\sim$33 s) for computing the zero-mean normalized cross-correlation (ZNCC) coefficient *Z*^40^.

**Figure 2:**
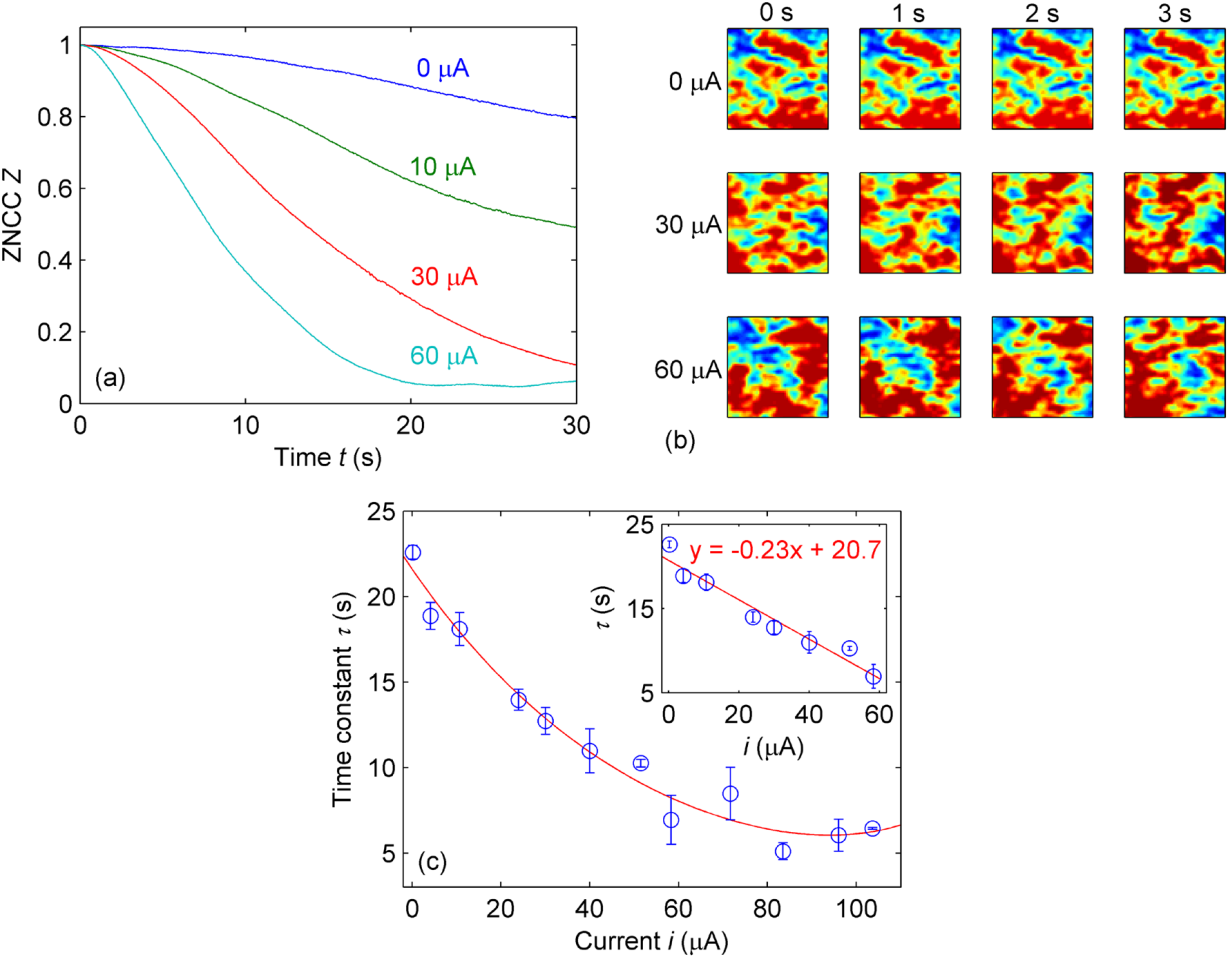
Optical response of agar spheres stimulated by electric current: (**a**) correlation coefficient as a function of time for different currents. (**b**) Temporal evolution of specklegrams, wherein colors indicate normalized intensity values. Original images cropped to $60 \times 60$ pixels ($0.28~\textrm{mm} \times 0.28~\textrm{mm}$) for the sake of viewing. (**c**) time constant of agar spheres as a function of the applied current. The solid line is a guide to the eyes. Inset: linear curve fitting for $0 \le i \le 60$ $\upmu$A ($R^2 = 0.950$).

Figure 2(a) shows *Z* as a function of time *t* for selected current values. The gradual decay of *Z* suggests that the specklegram changes become more active by increasing the magnitude of *i*. Figure 2b presents the speckle field intensity distribution *I*(*x*, *y*) for sequential acquisition frames and confirms such a premise. For example, *I*(*x*, *y*) remains unaltered during the initial seconds for $i = 0$ but progressively drifts for $i = 30$ and 60 $\upmu$A, proportionally reducing the correlation coefficient.

Assuming *Z*(*t*) as a sum of exponential decay functions^41^, one may normalize the ZNCC curves to compute their time constants $\tau$ at $Z(t = \tau ) \approx 0.368$. Figure 2c depicts $\tau$ versus *i*, where each data point comprises the average of five repetitions, and the error bars indicate the standard deviation of the mean and normal distributions with a probability level of 95%. Apart from the saturation for $i > 60$ $\upmu$A, the sensor response is essentially linear for $0 \le i \le 60$ $\upmu$A and presents a maximum deviation of $u_{\tau } \approx \pm 1.425$ s at 60 $\upmu$A, as shown in the inset of Fig. 2c, yielding an absolute sensitivity of $\textrm{d}\tau /\textrm{d}i \approx 0.233$ s/$\upmu$A with a practical resolution of $\Delta {}i \approx 1.425~\text {s}/0.233~\text {s.}\upmu \text {A}^{-1} \approx 6.116~\upmu$ A (or $\Delta {}i \approx 15^{-1}~\text {s}/0.233~\text {s.}\upmu \text {A}^{-1} \approx 0.286$ $\upmu$A considering the 15 Hz sampling rate of the acquisition system).

**Figure 3:**
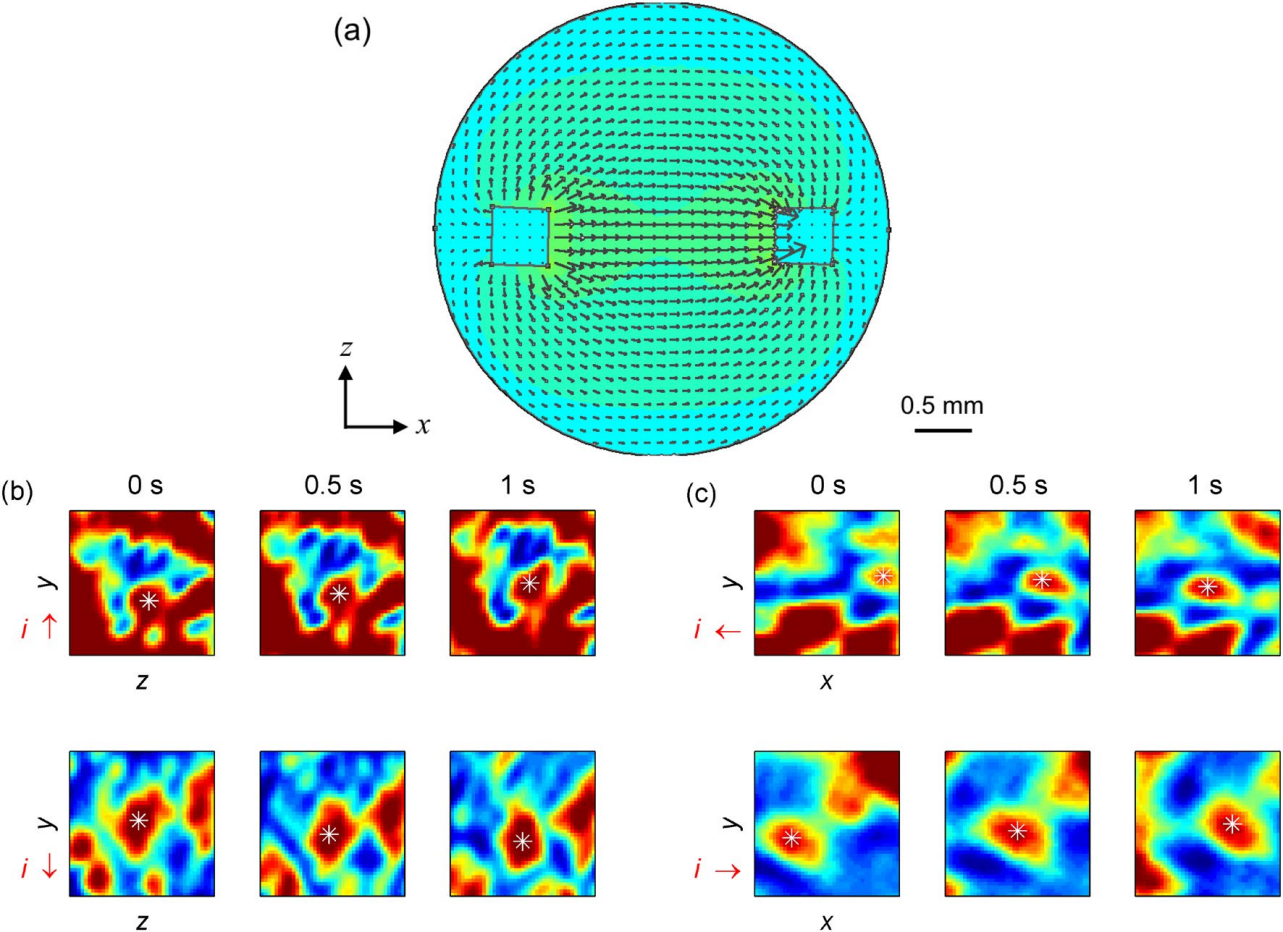
(**a**) Simulation of the electric field distribution inside the agar sphere with DC excitation through the pin headers. Colors indicate the electric field intensity. Spatiotemporal variation of specklegrams over the (**b**) *yz* and (**c**) *xy*-planes in response to currents *i* applied with different orientations (denoted by red arrows). The asterisks highlight the translation of an entity-of-interest. Colors indicate the normalized intensity values. Original specklegram images cropped to $40 \times 40$ pixels ($0.19~\textrm{mm} \times 0.19~\textrm{mm}$) for the sake of viewing.

As the specklegram temporal behavior varies with the magnitude of applied currents, the light granules may displace following the charges’ flow accordingly to the established electric field. Validation tests subjected the agar spheres to a constant current level of 60 $\upmu$A, while the orientation varied by transposing the electrodes parallel or perpendicularly to the light path, and the direction reversed by inverting the applied voltage. Figure 3a simulates the electric field distribution inside the agar sphere to predict how the current travels between the conductors. Given the coordinates system of Fig. 3a with light traveling towards the *z*-axis, an incident light beam parallel to the *x*-axis and striking the sphere above the pin headers would result in speckles moving vertically in the *yz*-plane, i.e., departing or arriving at the camera detection plane depending on the voltage source polarization. For example, speckles entering the image frame move downward to the pin header, whereas granules leaving the camera plane drift upward by departing the connector. Conversely, a perpendicular excitation would produce horizontal speckle motion once the current flow crosses the *xy*-plane. Figure 3b,c traces the displacement of light granules during 1 s to confirm this aspect. Altering the voltage source polarization also inverts the speckle pattern motion as predicted by simulation.

**Figure 4:**
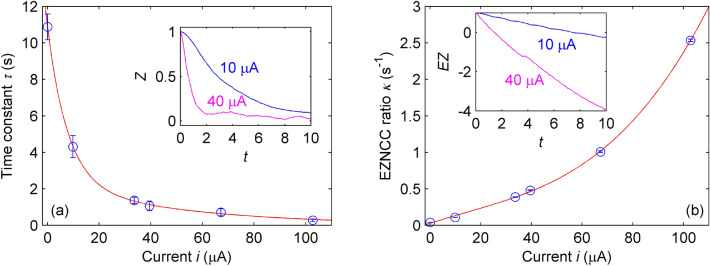
Optical response of agar waveguides. (**a**) Time constant $\tau$ as a function of the applied current *i*. Inset: ZNCC temporal evolution for $i = 10$ and 40 $\upmu$A. (**b**) EZNCC ratio $\kappa$ as a function of *i*. Inset: EZNCC temporal evolution for $i = 10$ and 40 $\upmu$A. The solid lines are guides to the eye.

Subsequent experiments investigated the current sensing capability of optical waveguides by replacing the spheres with no-core fibers (agar concentration of 2 wt% with diameter and length of 2.5 mm and 30 mm, respectively), as shown in Fig. 1. Light from a launching MMF couples the agar waveguide and generates the output speckle pattern *I*(*x*, *y*). After evaluating the ZNCC for 500 acquisition frames, the data processing routine normalizes the *Z*(*t*) curves and computes their time constants $\tau$, yielding the results of Fig. 4a, wherein data points comprise the average of five repetitions and the error bars assume the standard deviation of the mean with a 95% probability level. The ZNCC curves for agar fibers decay faster than those observed for spheres, as shown in the inset of Fig. 4a, reaching the saturation level for $i \ge 20$ $\upmu$A. The sensitivity within the linear range ($0 \le i \le 10$ $\upmu$A) is $\textrm{d}\tau /\textrm{d}i \approx 0.664$ s/$\upmu$A, corresponding to a practical resolution of $\Delta {}i \approx 0.671~\text {s}/0.664~\text {s.}\upmu \text {A}^{-1} \approx 1.011$ $\upmu$A given the maximum uncertainty of $u_\tau \approx \pm 0.671$ s at 0 $\upmu$A.

An alternative to circumvent the intrinsic saturation of the correlation function comprises evaluating the extended zero-mean normalized cross-correlation (EZNCC) score *EZ* to automatically reset the reference image $I_0$ whenever the output value reaches a threshold^42^. The inset of Fig. 4b portrays the EZNCC temporal evolution for 10 and 40 $\upmu$A to illustrate how the extended correlation translates the speckle field changes into a nearly linear behavior. Therefore, one may adopt the derivative of EZNCC $\kappa = |\textrm{d}EZ/\textrm{d}t|$ to quantify the specklegram deviations and compute its response to the input current. Figure 4b suggests a low-sensitivity range for $0 \le i \le 70$ $\upmu$ A with $\textrm{d}\kappa /\textrm{d}i \approx 0.015$ (s.A)$^{-1}$, followed by a gain-improved characteristic for $70 \le i \le 100$ $\upmu$A. The latter provides an absolute sensitivity of $\textrm{d}\kappa /\textrm{d}i \approx 0.043$ (s.A)$^{-1}$, resulting in a practical resolution of $\Delta {}i \approx 0.017~\text {s}^{-1}/0.043~\text {(s.}\upmu \text {A)}^{-1} \approx 0.395$ $\upmu$A for a maximum uncertainty of $u_\kappa \approx \pm 0.017$ s$^{-1}$ at 100 $\upmu$A.

## Discussion

Bulk agar samples work as scattering media due to their porous structure and the presence of microscopic bubbles, which explains the generation of speckle patterns by spheres despite their apparent transparency in the visible range. One may attempt to minimize the scattering centers by increasing the agar concentration and applying ultrasound/vacuum treatments to reduce the average pore size and collapse the air bubbles, respectively^43,44^. Besides, specklegrams manifest differently in no-core fibers once the waveguides confine the input light and promote interference between several guided modes. In this case, the fiber refractive index distribution, core size, and laser wavelength dictate the characteristics of light granules^45^.

Currents flowing through the agar-based devices promote spatially distributed temperature changes by Joule heating and generate refractive index deviations over the light path. Consequently, input currents modulate phase components of multiple guided modes and drift the output specklegrams. The optical devices remained stable during the experiments since this thermal increase cannot melt the solid agar *a priori*. Initial developments considered spheres instead of optical fibers because the former is easier to handle once coupled to the pin header connectors. Nevertheless, the results indicate equivalent behavior for no-core waveguides except for differences in the dynamic range, probably due to discrepancies in the dimensions of fiber and spheres.

Concerning the electrical conductivity $\sigma$ of agar-water samples, apart from the previous studies suggesting $0.05 \le \sigma \le 0.2$ S/m^24,28^, the actual values vary significantly for different agar manufacturers because of the mineral impurities^27^. Increasing the agar content ensures chemical stability and subtly raises $\sigma$, though concentrations $\le 2$ wt% are preferable to enhance the optical transmission in the visible range^19^. Alternatively, one may add glycerol to improve the agar devices’ transparency and mechanical strength with a moderate contribution to their electrical response^46,47^. Moreover, doping the agar-water solutions with salts augments the conductivity proportionally to their concentration^24,28,48^, which presumably boosts the specklegram sensitivity to input currents.

Regarding the fiber diameter and length, although these parameters influence the electrical resistance and, consequently, the voltage drop required for driving charges through the agar sample, the speckle field response to applied currents was consistent regardless of deviations in fiber geometries. Experiments assumed relatively short fibers as the optical losses ($\sim$3 dB/cm at 633 nm for agar-water samples) restrict their practical length^19^, yet improved transmission characteristics ($\sim$0.8 dB/cm) are still attainable by adding glycerol^31^ or eliminating microscopic air bubbles^44^.

The relative position of pin header connectors affects the current flow and may alter the speckle pattern dynamics depending on how the electric charges disturb the light path. The 2.5 mm gap between connectors (breadboard lead pitch) is enough to sustain an unbent $\sim$30 mm agar fiber and keep the optical system aligned during the electrical excitation, besides confining the current to a small volume. One may attempt enlarging this gap to flow currents throughout the waveguide extent, then amplify the optical modulation. However, the limited conductivity of agar-water samples demands a high threshold voltage to establish the electric potential, which may produce unwanted exceeding amperage peaks. The current range (0 to 100 $\upmu$A) considered safety values to not damage the sphere/fiber by overheating once preliminary tests with $i \le 300$ $\upmu$A indicated rapid probe deterioration as the bulk agar melted.

Tracking the speckle granules’ motion due to alternate currents is plausible but depends on the capabilities of the data acquisition system. For example, experimental setups for mechanical vibration sensing achieve the temporal resolution of a few $\upmu$s by employing a high-speed camera and fast speckle pattern processing^49^. Assuming AC signals of $< 100$ Hz, moderate video sampling rates ($\le 30$ Hz) cannot track the alternating speckles but are potentially sensitive to the root-mean-square value of input currents. Furthermore, 2D image matching techniques, such as the phase-only correlation^40^, can simultaneously retrieve the magnitude and direction of applied stimuli.

The EZNCC algorithm prevents the correlation coefficient from saturating, which virtually extends the measurement range beyond 100 $\upmu$A. However, the heat flow through the optical fiber becomes critical as the electrical conductivity of agar increases with the temperature^27^, softening the waveguide structure and yielding optical losses. The agar spheres also presented a darkened region between the pin headers in response to an exceeding voltage drop. Alternatively, enlarging the fiber diameter or the gap between conductors could ameliorate the thermal distribution and detain the agar degradation.

One may notice that the agar devices gradually shrink under room conditions due to syneresis^23^, causing specklegram changes even for undisturbed conditions, as noticed in Fig. 2a. Conserving the waveguides with plastic wrap before the experiments impedes the agar from expelling water droplets and preserves its structure. Avoiding variations in environmental temperature and pH also guarantees chemical stability^13,23^. Nevertheless, this effect is less prominent, though not negligible, in agar-glycerol-water compositions, yielding biodegradable fibers that endure for weeks^46^. Regarding the humidity, water diffuses through the bulk agar structure and increases its volume^23^. Besides swelling, changes in the water content also reduce the electrical conductivity by diluting the mineral impurities. Thus, one may expect the simultaneous contribution of humidity and current in the output specklegram. Albeit discerning both effects is not straightforward, Fig. 3 suggests that the speckle granules consistently follow the current flow, i.e., tracing their displacement rate through image registration and active contouring algorithms^50^ yield an estimation of the electrical stimulus regardless the agar volumetric changes.

Concerning the interrogation system, launching silica or polymer fibers are preferable to interface the agar probe to the measurement apparatuses once the critical optical losses prevent the implementation of an all-biodegradable fiber setup^2^. For example, one may use a pair of emitting and collecting waveguides to guide the light through the agar sphere or fiber, establishing a single-path configuration. However, speckle patterns generated by launching and sensing waveguides are vulnerable to extraneous variables like vibration and temperature, demanding reference fibers or software-based corrections for practical operation^51^. Some applications also require a reflection-type probe to isolate the camera from the current source and provide non-invasive assessments, i.e., attaching a mirror to the fiber end face and deriving the reflected speckle with a multimode fiber coupler^52^. Besides, perforating the agar waveguides may be unfeasible for real applications, which demand detecting the currents flowing through the surface rather than inside the bulk agar. No-core fibers are sensitive to surrounding stimuli and can perceive the refractive index changes imposed by the outer electric charges a priori. Nevertheless, grounding the agar structure is fundamental for establishing currents to modulate the output specklegram.

Notwithstanding its merits, the proposed sensor exhibits a limited dynamic range compared with typical optical fiber sensors based on strain gauges with magnetostrictive coatings^34,36^, holey fibers filled with ferrofluids^33^, and polarization-type, Faraday rotation transducers^35^. The current constraint of 100 $\upmu$A frustrates applications in high-powered systems but endorses the ability to detect bioelectrical signals^37,38^. Furthermore, agar waveguides are eligible for substituting biodegradable electrodes based on expensive materials and complex structures^53^.

In conclusion, this paper proposed an all-optical current sensor employing agar-based waveguides and speckle field processing. We exploited the electrical conductivity of agar-water samples to modulate the transmitted light and detect the magnitude and direction of input currents for the first time in the literature. Besides its future uses in biomedical analyses, one may envisage electrically driven, active optical devices for light control applications, i.e., shaping the refractive index distribution of the agar device through the applied electric field, then explore the speckle field characteristics to select specific modes in data transmission and space-division multiplexing systems or clean the speckled output as a non-vibrating mode scrambler^54,55^. Several challenges persist regarding the measurement range, noise, and reliability, which inspire further developments for the agar-based fiber design and the optical interrogation system to attain practical biodegradable sensors.

## Methods

### Fabrication of agar samples

Solutions containing food-grade agar (non-sterile, ash content $\le 6.5$%) and distilled water heat under constant agitation provided by a hot plate equipped with magnetic stirrer. After reaching the boiling point, the mixture settles at room temperature for $\sim$2 min to collapse air bubbles. A silicone tubing (inner diameter of 2.5 mm) serves as the mold to fabricate cylindrical waveguides. Once a syringe completes the tubing with melted agar, the solution solidifies in a refrigerator for $\sim$15 min. Finally, the experimentalist expels the fiber from the mold and cleaves the end faces using a razor blade^31^.

Regarding the spheres, a transfer pipette drips the melted agar on food-grade soybean oil subjected to slow circulation at room temperature. The agar drops solidify into spheres with an average diameter of 4 mm. Subsequently, the samples are washed with distilled water, sieved, and cleaned in the ultrasonic bath to remove oil droplets impregnated in the agar surface.

**Figure 5:**
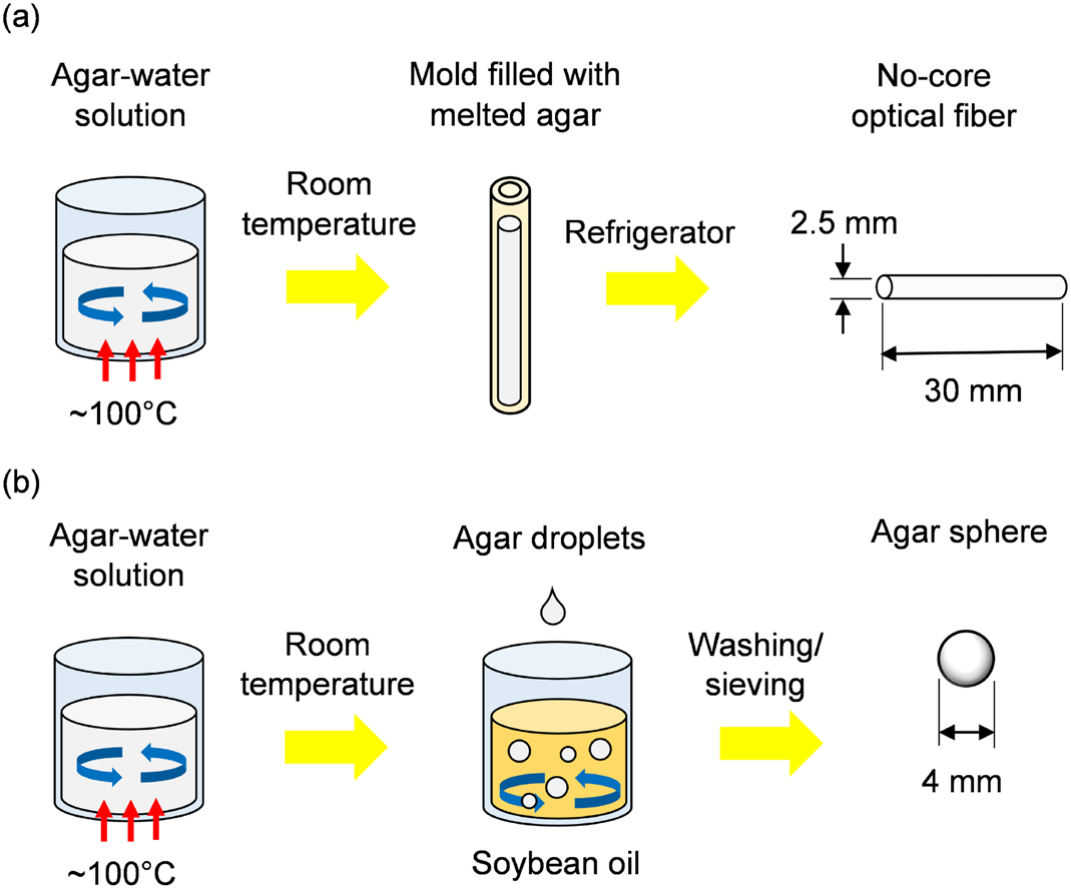
Fabrication of (**a**) agar-based no-core fibers and (**b**) spheres for electric current sensing.

Figure 5a and b summarize fabrication procedures of agar-based optical fibers and spheres, respectively.

### Measurement setup

**Figure 6:**
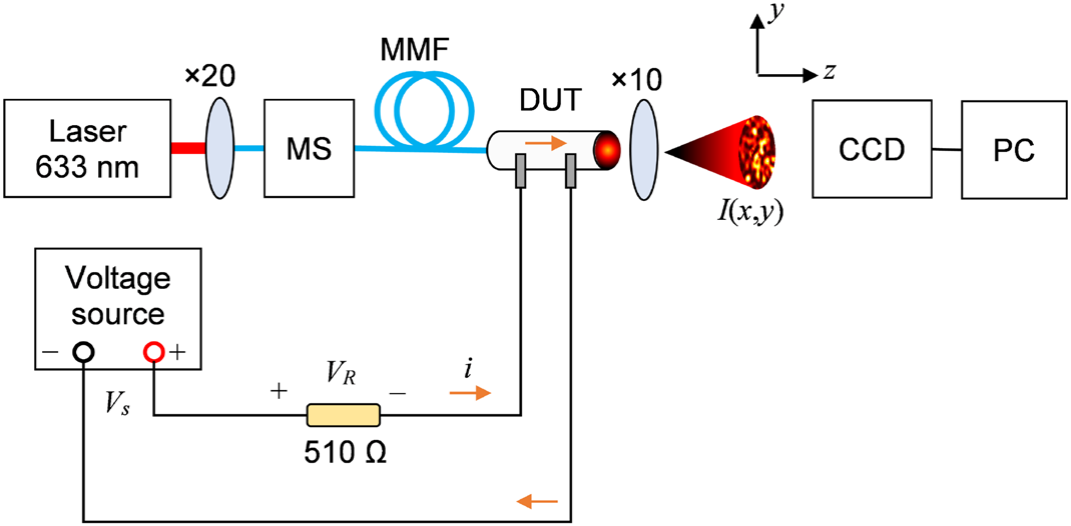
Measurement setup. The HeNe laser illuminates the agar device under test (DUT), producing an output speckle pattern *I*(*x*, *y*) detectable by the CCD camera. Meanwhile, the DC voltage source $V_s$ excites the circuit and drives a current along the DUT to electrically modulate *I*(*x*, *y*).

The experiments proceed in a dark environment at room temperature with the apparatuses portrayed in Fig. 6 mounted on a vibration-free table. A HeNe laser (wavelength of 633 nm with 1 mW power) illuminates a launching silica multimode fiber (MMF, step-index, core and cladding diameters of 62.5 $\upmu$m and 125 $\upmu$m, respectively) through a $20\times$ objective. After passing a mode scrambler (MS) for equalizing the modal power distribution, the MMF couples the biodegradable optical device by carefully introducing the MMF tip inside the agar sample through a micrometric stage. Then, a lensless CCD camera ($1024\times 768$ pixels resolution, pixel size of 4.65 $\upmu$m, and detector size of 4.8 mm$\times$3.6 mm) acquires the output speckle patterns magnified by a $10\times$ objective at a 15 Hz sampling rate and delivers the data to the computer for further processing by MATLAB (Mathworks) routines.

A DC voltage source $V_s$ (output range of 0 to 32 V, 10 mV resolution) connected to an $R = 510 \Omega$ reference/protection resistor generates controlled currents for perturbing the optical signal. The agar device connects in series to the circuit with a pair of standard male pin headers mounted onto a breadboard. The pin headers partially perforate the waveguide without occluding the light path, creating a gap of $\sim$2.5 mm between the conductors. Lastly, a digital multimeter (voltage and current resolutions of 0.01 mV and 0.1 $\upmu$A, respectively) monitors the voltage drop $V_R$ across *R* to estimate the current flowing through the agar sample.

The tests involved five spheres/fibers excited with increasing voltages, yielding five specklegram videos per input current condition. The samples remained in distilled water to conserve their structure, and the experimentalist carefully dried the agar devices with a paper towel to remove the excess liquid before the measurements, discarding the perforated materials after their application. It is worth noticing that the agar samples remained stable for more than 1 week.

### Specklegram image processing

The MATLAB routine extracts the input video frames, converts the speckle field images from RGB space to grayscale, and crops square regions of interest comprising $201 \times 201$ pixels. Let *I*(*t*) the specklegram distribution over the *xy*-plane at time *t*, and $I(0) = I_0$ the reference state. Speckle pattern deviations induced by the electrical stimulus are quantifiable by computing the zero-mean normalized cross-correlation (ZNCC) coefficient *Z*(*t*),

1 $$ Z(t) = \frac{{\iint {(I - \bar{I})}(I_{0}  - \bar{I}_{0} ){\text{d}}x{\text{d}}y}}{{\left[ {\iint {(I - \bar{I})^{2} }{\text{d}}x{\text{d}}y\iint {(I_{0}  - \bar{I}_{0} )^{2} }{\text{d}}x{\text{d}}y} \right]^{{1/2}} }}, $$

the overlines denote the average values of *I* and $I_0$^40^.

$Z(t) = 1$ when $I = I_0$ and its value gradually decreases as the fiber state departs from the reference condition so that the decorrelation becomes more abrupt as the speckle changes become vigorous^56,57^. Given the heterogeneous nature of moving speckle granules, one may approach the ZNCC temporal evolution to a sum of *N* exponential decay functions $Z(t) = A + \sum _{n=1}^{N} B_n \exp (-t/\tau _n)$ denoted by their coefficients *A* and $B_n$, and take the first (slowest) term $\tau _1$ as the average decay rate ${\bar{\tau }}$ of the *N* exponential functions^41^. In this sense, the algorithm normalizes the *Z*(*t*) curves and evaluates the time constant $\tau = {\bar{\tau }}$ relative to $Z(\tau ) = e^{-1} \approx 0.37$.

Once the ZNCC saturates as the differences between *I* and $I_0$ increase, the MATLAB routine also evaluates the extended zero-mean normalized cross-correlation (EZNCC) coefficient *EZ*(*t*),

2 $$\begin{aligned} EZ(t) = EZ_0 - \left[ Z(t,I,I_0,\alpha ) -1 \right] , \end{aligned}$$

wherein $EZ_0$ is a cumulative offset, *Z*(*t*) is the ZNCC evaluated as a function of the intensities *I* and $I_0$, and $\alpha = 0.8$ is an empirically chosen threshold. This procedure begins with $EZ(t) = Z(t)$ and follows the correlation curve until $Z(t) < \alpha$. At this condition, the algorithm resets the reference state making $I_0 = I(t)$ and $EZ_0 = EZ(t)$, thus, preventing the EZNCC saturation and converting the exponential decay behavior of *Z*(*t*) into a practically linear decrease^42^. Consequently, one may quantify the specklegram temporal changes as the derivative of EZNCC $\kappa = \textrm{d}EZ/\textrm{d}t$ as a counterpart to the ZNCC time constant.

### Electric field simulation

Simulations proceeded with the free software FEMM (Finite Element Method Magnetics)^58^, assuming an electrostatic problem to trace the field lines along the *xz*-plane of the agar sphere. The brass electrodes comprise 0.5 mm$\times$0.5 mm squares spaced by 2 mm with electrical permittivity of $\epsilon = 1$ F/m. The sphere has a diameter of 4 mm and $\epsilon = 80.4$ F/m (approximating the $\epsilon$ value of 2 wt% agar to the water one). Thus, the software computes the electric field density distribution for a DC voltage drop of 30 mV and mesh grid size of 0.1 mm.

## Data Availability

Datasets and original images are available from the corresponding author on request.
